# *IL-18* Gene rs187238 and rs1946518 Polymorphisms and Expression in Gingival Tissue in Patients with Periodontitis

**DOI:** 10.3390/biomedicines10102367

**Published:** 2022-09-22

**Authors:** Małgorzata Mazurek-Mochol, Magdalena Brzeska, Karol Serwin, Damian Malinowski, Krzysztof Safranow, Edyta Zagrodnik, Iwona Stecewicz, Andrzej Pawlik

**Affiliations:** 1 Department of Periodontology, Pomeranian Medical University, 70-204 Szczecin, Poland; 2 Department of Physiology, Pomeranian Medical University, 70-204 Szczecin, Poland; 3 Department of Pharmacology, Pomeranian Medical University, 70-204 Szczecin, Poland; 4 Department of Biochemistry and Medical Chemistry, Pomeranian Medical University, 70-204 Szczecin, Poland; 5 Department of Anestesiology, Pomeranian Medical University, 70-204 Szczecin, Poland; 6 Department of Pediatrics, Pomeranian Medical University, 70-204 Szczecin, Poland

**Keywords:** IL-18, gene, polymorphism, periodontal disease, periodontitis, gingival tissue, expression

## Abstract

Periodontitis is a chronic disease with disturbed balance between the immune and inflammatory response of the host to bacteria. Many studies have shown that proinflammatory cytokines play a significant role in the pathogenesis of periodontal disease. In this study, we examined the association between the *IL-18* gene rs187238 and rs1946518 polymorphisms and periodontitis in non-smoking and smoking patients. This study enrolled 200 patients with periodontitis (130 non-smokers and 70 smokers) and 156 control subjects (124 non-smokers and 32 smokers). There were no statistically significant differences in the distribution of the rs187238 and rs1946518 IL-18 genotypes and alleles between patients with periodontitis and control subjects, between smoking patients with periodontitis and smoking control subjects, and between non-smoking patients with periodontitis and non-smoking control subjects. There were no statistically significant differences in clinical parameters in relation to the *IL18* rs187238 genotypes. In patients with the *IL18* rs1946518 GG genotype, we observed increased values of bleeding on probing (BoP) and periodontal probing depth (PPD), compared to subjects with the TT genotype. In patients with periodontitis, we observed statistically significant decreased expression of the IL-18 gene in comparison with healthy subjects (0.231 ± 0.163 vs. 0.663 ± 0.197, *p* = 0.0008). In addition, the IL-18 gene expression in gingival tissue in patients with periodontitis correlated positively with the number of remaining teeth. The results of our study suggest that the IL-18 rs187238 and rs1946518 polymorphisms are not significant risk indicators of periodontitis in our population. However, in patients with the *IL18* rs1946518 GG genotype, we observed increased values of BoP and PPD, compared to subjects with the TT genotype. In addition, in gingival tissue of patients with periodontitis, we have detected decreased expression of IL-18. The gingival expression of IL-18 in patients with periodontitis correlated positively with number of remaining teeth. The above results suggest that IL-18, in addition to its pro-inflammatory effects in periodontal disease, may also exhibit protective properties.

## 1. Introduction

Periodontitis is a chronic inflammatory disease caused by an imbalance between the immune and inflammatory responses to bacterial infection. The immune response stimulates the production of inflammatory mediators, such as cytokines and chemokines, which induce inflammation in periodontal tissues, leading to their destruction and tooth loss [1,2,3]. The pathogenesis of periodontitis is multifactorial and has not been fully understood. It is believed that the development of periodontal disease depends on the influence of numerous environmental, microbiological, and genetic factors that can modulate the immune response [4,5]. Risk factors are most commonly divided into modifiable and non-modifiable factors. Non-modifiable factors include age, gender, race, and genotype. Modifiable ones include poor oral hygiene, diabetes, osteoporosis, and smoking. Tobacco smoking is an important environmental risk factor for the development of periodontal disease [6]. Smoking has been shown to modulate immune response and increase the risk of the development and progression of periodontitis by up to 85%, as well as negatively affect the results of periodontal treatment [1]. Although the association of smoking with periodontitis is well supported by clinical studies, unfortunately, the exact mechanisms involved still have to be defined [7]. It has been shown that various substances in tobacco smoke negatively affect blood vessels, inducing vasoconstriction and inhibiting angiogenesis [7]. Previous studies have examined the effect of genes on periodontal disease in both smoking and non-smoking patients. 

Periodontal disease often leads to tooth loss, so genetic factors predisposing to its development are being sought. Among them are genetic polymorphisms in genes that regulate the synthesis of pro-inflammatory cytokines. Numerous studies have examined the association between cytokine gene polymorphisms and periodontal disease, but the results are inconsistent. One reason for the observed discrepancies may be differences in the classification of periodontal disease. The American Academy of Periodontology and the European Federation of Periodontology at the World Workshop in November 2017 in Chicago presented a new classification of periodontal diseases and tissues around implants, in which forms of the disease previously considered “chronic” or “aggressive” are now grouped into a single category—“periodontitis” [3,8]. The new classification from 2017 distinguishes three forms of periodontitis: periodontitis (previously divided into chronic and aggressive), necrotizing periodontitis, and periodontitis as a manifestation of a systemic disease. This classification depends largely on the severity of the disease at the time of onset, as well as its complexity. This classification provides additional information on the biological characteristics of the disease, such as estimated rate of disease progression based on its current course, risk of further progression, anticipated treatment failure, and assessment of the risk that the disease or its treatment may adversely affect the general health of the patient [8].

In periodontal tissue, various factors induce the immune response, leading to the production of inflammatory mediators, such as pro-inflammatory cytokines. Many studies have shown that pro-inflammatory cytokines play a significant role in the pathogenesis of periodontal disease [4,5,9]. 

Interleukin-18 is a cytokine produced by various hematopoietic and nonhematopoietic cells [6]. This interleukin has chemotactic and angiogenic properties and could play an important role in the development of periodontal disease [10,11]. One of the main functions of IL-18 is to enhance interferon-γ production in the presence of IL-12 and promote Th1 responses. However, IL-18 also enhances Th2 responses and the synthesis of other pro-inflammatory cytokines, such as TNF, IL-1β, and IL-8, as well as granulocyte colony-stimulating factor and macrophage colony-stimulating factor, which are involved in inflammatory process in periodontitis. Dysfunction of the Th1/Th2 immune response leads to inflammation and progression of periodontal disease [5]. Previous studies have shown that IL-18 is involved in a number of immune processes, leading to the development of periodontitis [12,13,14]. High levels of IL-18 are detected in inflammatory and autoimmune diseases, such as rheumatoid arthritis, psoriasis, and systemic lupus erythematosus [5,14,15,16].

IL-18 protein production is regulated by the IL-18 gene. The human IL-18 gene is located on chromosome 11q22.2-22.3 [9,17]. In the IL-18 gene, several functional polymorphisms were detected, which can change the IL-18 gene expression and IL-18 synthesis [18]. Two single-nucleotide polymorphisms (SNPs), rs187238 and rs1946518, have been widely studied in patients with inflammatory diseases. These two SNPs are located within a transcription factor-binding element, thereby influencing transcription from the *IL-18* gene [19]. 

The aim of the study was to examine the *IL-18* rs187238 and rs1946518 polymorphisms in patients with periodontitis, in relation to smoking habits, as well as the expression of IL-18 in gingival tissue of patients with periodontitis and in healthy subjects.

## 2. Materials and Methods

### 2.1. Study Subjects

This study enrolled 356 Caucasian subjects (age range 25–75 years) from the West Pomeranian region of Poland. The subjects were submitted to anamnesis and to clinical and periodontal examination. The subjects were divided into two subgroups: patients with periodontitis and healthy subjects without periodontitis. The first group comprised 200 patients (84 men, 116 women), aged 26–75 years (mean 49.85 ± 8.71), with periodontitis, diagnosed using the 2017 classification system of periodontal diseases [3,8]. The patients with diagnosed periodontitis were defined if loss of interdental attachment level (AL) ≥ 2 mm was detectable at two or more non-adjacent teeth, or buccal or oral AL ≥ 3 mm and periodontal pockets > 3 mm were detectable at two or more teeth, and the observed AL could not be attributed to non-periodontal causes [8]. Radiographic bone loss had to be at least 15%. Extent and distribution of periodontitis was described as generalized when more than 30% of teeth were involved. 

Out of group of 200 patients with periodontitis, 130 were non-smokers and 70 were smokers. The control group consisted of 156 subjects (54 men and 102 women, aged 25–74 years; mean: 45.40 ± 10.18). According to the actual classification, periodontally healthy subjects have no history of periodontitis, no radiographic evidence of bone loss, no sites with a probing depth > 3 mm, no interdental AL, and a full-mouth bleeding score < 10%. In the healthy group, 124 subjects were non-smokers and 32 were smokers. 

Exclusion criteria included systemic disease, patients who used systemic or subgingival antimicrobial agents, or were chronic users of anti-inflammatory medication. Subjects were also excluded from the study if they had a history of hepatitis, AIDS or HIV, recent radiation therapy, diabetes, uncontrolled hypertension, use of immunosuppressive medications, or were pregnant.

The subjects were categorised into four subgroups: smoking and non-smoking with periodontitis, and smoking and non-smoking controls. Patients who had smoked tobacco were allocated to the smoking group with or without periodontitis. Patients who had never smoked were placed in the non-smoking group with or without periodontitis. 

At the initial stage of treatment in patients with periodontitis, professional mechanical plaque removal (PMPR) was performed from supragingival surfaces. Subgingival instrumentation with antibacterial agents (chlorhexidine) was then performed. Instruments such as piezoelectric or ultrasonic scalers were used in combination with manual instruments (curettes). After subgingival instrumentation with antibacterial treatment, 162 patients achieved clinical improvement. Nineteen patients who did not achieve improvement were treated surgically with open flap debridement. In patients who underwent open flap debridement (OFD), the area to undergo surgery was anaesthetized with a 4% articaine hydrochloride solution with adrenaline (1: 100,000), intracrevicular incisions were placed and the granulation tissue was removed from the defects, the roots were scaled and planed, and then the gums were sutured with simple interrupted sutures. In 14 patients from this group, sections of the gingival tissue were taken for assessment of IL-18 expression. The control group consisted of 14 healthy subjects without periodontal disease who had minor oral surgery (crown lengthening for prosthetic indications or extraction due to orthodontic grounds).

The study was approved by the ethics committee (BN-001/93/08) at Pomeranian Medical University, Szczecin, Poland, and written informed consent was obtained from all subjects.

### 2.2. Periodontal Examination

Periodontal evaluation included periodontal probing depth (PPD), clinical attachment level (CAL), the approximal plaque index (API), and bleeding on probing (BoP). Both PPD and CAL were assessed at six sites per tooth (disto-buccal, mesio-buccal, midbuccal, disto-lingual, mesio-lingual/palatal, and mid-lingual/mid-palatal). Pocket depth represents the distance from the gingival margin to the bottom of the periodontal pocket and CAL represents the distance from the cemento-enamel junction to the bottom of the periodontal pocket. API was assessed based on the presence of plaque in the interproximal surfaces (quadrant—Q1 and 3—oral, Q2 and 4—facial aspect) and was calculated using the following formula: (no. of plaque (+) sites/no. of sites examined) × 100. A bleeding on probing (BoP) score was assessed as the proportion of bleeding sites (dichotomous yes/no evaluation) when probing the sulcus/pocket at six sites on all present teeth. A UNC-15 colour-coded probe (Hu-Friedy Mfg Co. Inc., Chicago, IL, USA) was used for all explorations. 

### 2.3. Genotyping

Genomic DNA was extracted from whole blood collected in EDTA tubes using a GeneMATRIX Quick Blood DNA Purification Kit (EURx, Gdansk, Poland). Identification of all examined polymorphisms in *IL18* gene was performed using the TaqMannSNP genotyping assay (Applied Biosystems, Carlsbad, CA, USA): rs187238 (C___2408543_10) and rs1946518 (C___2898460_10). The reaction was performed in duplicate on a 7500 Fast Real-Time PCR Detection System (Applied Biosystems). 

### 2.4. RNA Isolation

Tissue samples (50–100 mg) were suspended in 600 µL of RLT buffer (Qiagen, Germany) and homogenized using Ultra-Turrax T-10 basic (IKA^®^) dispersing tool (4 min at 30,000 rpm/min). Disintegrated samples were digested using proteinase K (10 min at 55 °C). Total RNA was extracted using an RNeasy Lipid Tissue Mini Kit (Qiagen, Germany), according to the manufacturer’s protocol. The concentration and purity of RNA samples was determined by measuring the absorbance using a spectrophotometer NanoDrop ND-1000 (NanoDrop Technologies, Wilmington, DE, USA). Next, cDNA was prepared from 0.5 µg of total cellular RNA in a 20 µL reaction volume, using a RevertAid First Strand cDNA synthesis kit (Thermo Scientific, Waltham, MA, USA), according to the manufacturer’s protocol. 

### 2.5. Real-Time Quantitative Reverse Transcription Polymerase Chain Reaction (RQ-PCR)

Quantitative assessment of the mRNA levels was performed by RQ-PCR using an ABI 7500 Fast instrument (Applied Biosystems, USA) with Power SYBR Green PCR Master Mix reagent (Applied Biosystems, USA). Real-time conditions were as follows: 95 °C (15 s), 40 cycles at 95 °C (15 s), and 60 °C (1 min). Specificity assessment was done by performing melting curve analysis (only one PCR product was amplified under these specific conditions). Each sample was analysed in duplicate, and the mean cycle threshold (Ct) values were used for further analysis. To calculate the values, the 2^−ΔCt^ method was used. Obtained data were normalized to β2-microglobulin. The sequences of used primers were prepared according to the sequence information obtained from the NCBI database, and were synthesized by Oligo.pl (IBB PAN, Warszawa, Poland). 

### 2.6. Statistical Analysis

The consistency of the genotype distribution with Hardy–Weinberg equilibrium (HWE) was assessed with Fisher’s exact test. Chi-square and Fisher’s exact tests were used to compare the genotype and allele distributions between study groups. 

Distributions of quantitative variables significantly differed from a normal distribution (Shapiro–Wilk test); thus, non-parametric tests were used. Values were compared between genotype groups with a Kruskal–Wallis or Mann–Whitney test, and correlations within groups were assessed with a Spearman rank correlation coefficient. A statistically significant result was considered *p* < 0.05. This study, with 200 patients and 156 controls, had sufficient statistical power to detect, with 80% probability, the real allelic associations, with a strength corresponding to an odds ratio (OR) of 0.60 or 1.59 for *IL18* rs187238 and 0.64 or 1.55 for *IL18* rs1946518.

## 3. Results

The baseline clinical periodontal parameters in the studied groups are shown in Table 1. 

The first step of our study was to compare the distribution of the IL-18 gene polymorphisms between patients with periodontitis and healthy subjects to see if these polymorphisms are risk indicators of periodontitis. The distribution of the *IL-18* rs187238 and rs1946518genotypes among patients with periodontitis and control subjects was in HWE and is shown in Table 2. There were no statistically significant differences in the distribution of the *IL-18* rs187238 and rs1946518 genotypes and alleles between patients with periodontitis and control subjects (Table 2).

We also compared the distribution of the studied polymorphisms between smoking patients with periodontitis and smoking control subjects, and between non-smoking patients with periodontitis and non-smoking control subjects. As shown in Table 3 and Table 4, these differences were not statistically significant.

The next point of our work was to evaluate whether the studied polymorphisms of the IL-18 gene affect clinical parameters in patients with periodontitis. We compared the clinical parameters in patients with periodontitis between different genotypes. There were no statistically significant differences in the clinical parameters in relation to the *IL18* rs187238 genotypes. In patients with the *IL18* rs1946518 GG genotype, we observed increased values of BoP and PPD, compared to subjects with the TT genotype (Table 5 and Table 6). There was also no association between the polymorphisms studied and the results of the periodontitis treatment (Table 7 and Table 8).

**Figure 1:**
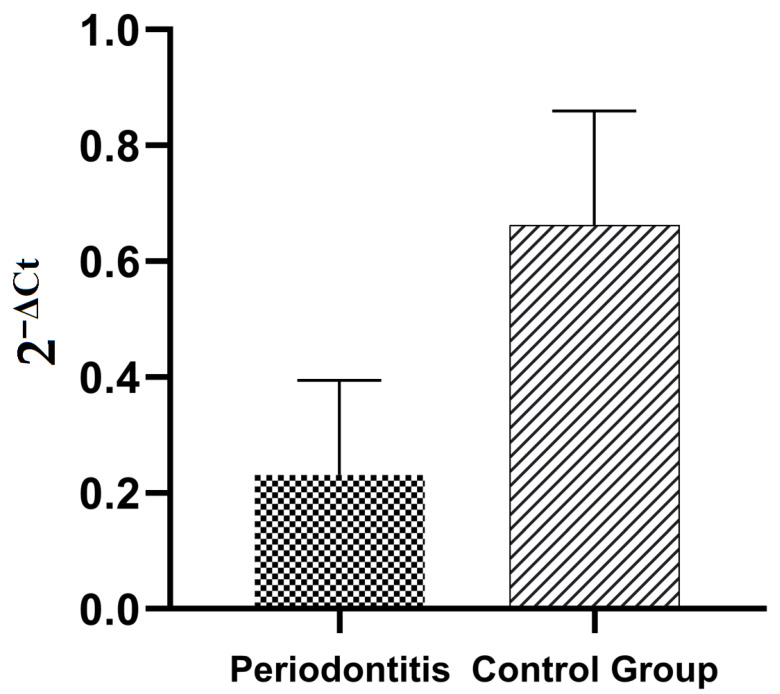
IL-18 expression in gingival tissue in patients with periodontitis and the control group, *p*-0.0008.

Another point of our study was to examine IL-18 expression in gingival tissue and to correlate IL-18 expression in gingival tissue with clinical parameters in patients with and without periodontitis. In patients with periodontitis, we observed statistically significant decreased expression of the IL-18 gene in comparison with healthy subjects (0.231 ± 0.163 vs. 0.663 ± 0.197, *p* = 0.0008) (Figure 1). In addition, the IL-18 gene expression in gingival tissue in patients with periodontitis correlated positively with the number of remaining teeth (Table 9). In the control group there were no statistically significant correlations between the IL-18 expression in gingival tissue and clinical parameters (Table 10).

## 4. Discussion

In this study, we examined the associations between the *IL-18* rs187238 and rs1946518 polymorphisms and periodontitis. Our results suggest that these polymorphisms are not significant risk indicators of periodontitis development, both in smoking and non-smoking patients in our population. We also examined the associations between the studied polymorphisms and clinical parameters in patients with periodontitis. There were no statistically significant differences in clinical parameters in patients with different IL*-18* rs187238 genotypes. Conversely, in patients with the *IL-18* rs1946518 GG genotype, we observed increased values of BoP and PPD, compared to subjects with the TT genotype. In addition, we examined the expression of the *IL-18* gene in the gingival tissue. The expression of the *IL-18* gene in patients with periodontitis was significantly decreased in comparison with healthy subjects. Moreover, the *IL-18* gene expression of gingival tissue in patients with periodontitis correlated positively with the number of remaining teeth. 

Previous studies investigated the IL-18 gene polymorphisms as the factors influencing the risk of periodontitis development and its course; however, the results are inconsistent. Folwaczny et al. [20] examined six IL-18 gene polymorphisms in patients with destructive periodontal disease in a German population. These authors did not find associations between IL-18 gene polymorphisms and destructive periodontal disease. The results in Noack et al. [21,22] also suggest lack of association between IL-18 gene polymorphism and periodontal disease in the German population. Similarly, in the studies by Vokurka et al. [23] and Borilova Linhartova et al. [24], there was no statistically significant association between IL-18 gene polymorphism and periodontal disease in a Czech population. Martelli et al. [25] found a moderate association between IL-18 gene polymorphisms and chronic and aggressive periodontal disease in an Italian population. The results by Tanaka et al. [26] suggest the association between the IL-18 promoter rs1946518 polymorphism and periodontitis in young Japanese women. Shan et al. have found the association between IL-18 gene polymorphisms and susceptibility to periodontitis in a Uyghur population [27]. In a Brazilian population, an association was found between IL-18 (rs187238 and rs1946518) and chronic periodontitis [28]. The meta-analyses by Li et al. and da Silva et al. suggest that IL-18 gene polymorphisms are associated with increased risk of periodontitis [9,29]. Therefore, the association between IL-18 rs187238 and rs1946518 polymorphisms and periodontal disease remains controversial. The differences observed in studies may be caused by environmental and ethnic differences. In various populations these polymorphisms may be linked with different polymorphisms in genes involved in production of other proinflammatory mediators, such as cytokines and chemokines. Moreover, other factors may influence production of IL-18. In some populations, pathogenic microflora and other environmental factors, such as diet and smoking, may exert a more significant effect on IL-18 production than genetic polymorphisms. Previous studies have also shown that the influence of genetic polymorphisms on the risk of periodontal disease may depend on smoking habits. Smoking is a factor that can significantly affect the onset of periodontal disease, its clinical course, and the effectiveness of treatment. Smoking can also mask early signs and symptoms of periodontal disease by reducing the clinical signs of gingival inflammation. To date, there are no specific preventive and periodontal therapies for smokers. Smoking cessation is the best practicable way to reduce the risk of periodontitis onset and progression and improve treatment outcomes [30,31].

Periodontal disease is a chronic inflammatory disease induced by various proinflammatory mediators, such as cytokines and chemokines. IL-18 is the cytokine produced by various cells, especially cells of immune systems (macrophages, T cells, NK cells, NKT cells, B cells, and dendritic cells), but also by other cells, including keratinocytes, fibroblasts, epithelial cells, and osteoblasts. There are many studies examining the role of IL-18 in periodontal pathogenesis, indicating that its action may be multidirectional [3,11,12,13,14,15,32,33].

The majority of previous studies showed increased levels of IL-18 in the crevicular fluid and saliva of patients with various forms of periodontal disease [34,35,36,37,38,39], whereas the expression of IL-18 in gingival tissue biopsy specimens did not differ significantly between patients with periodontal disease and healthy subjects, or was even decreased in some forms of periodontitis [40,41]. However, there are also studies indicating decreased levels of IL-18 in gingival crevicular fluid in patients with gingivitis. Keles et al. have also shown that gingival crevicular fluid levels of IL-18 in children with gingivitis were significantly lower than in periodontally healthy children [42]. 

Our study found a decreased expression of IL-18 in gingival tissue in patients with periodontitis. Moreover, we found that in patients with periodontitis, the expression of IL-18 correlates with the number of remaining teeth, reflecting the progression and severity of the disease process. However, this raises the question of why the reduced IL-18 expression in gingival tissue of patients with periodontitis was observed. There could be several explanations for this. Tissue specimens were collected during periodontal surgery, in patients who had previously undergone non-surgical periodontal treatment, which may have affected IL-18 expression. Decreased IL-18 expression in the gingival tissue of periodontitis patients may also be a result of chronic inflammation and progression of the disease process. This seems to be confirmed by the positive correlation between IL-18 expression in gingival tissue and the number of remaining teeth. It is likely that patients with an advanced disease process and chronic inflammation have reduced IL-18 expression in gingival tissue due to the influence of other pro-inflammatory mediators. It cannot be excluded that, in periodontitis patients, IL-18 may have some beneficial effects. It has been shown that IL-18 stimulates osteoblast proliferation and promote osteoblastogenesis [43,44]. Osteoblasts, which are bone-forming cells, play crucial roles in bone modelling and remodelling. Osteoblasts play a key role in bone formation. Disruption of their function can lead to diseases such as rheumatoid arthritis, osteoporosis, and periodontal disease. Our observation of a positive correlation between IL-18 expression and the number of remaining teeth in periodontitis patients may suggest that IL-18 may not only have pro-inflammatory effects in periodontitis, but by stimulating the process of osteogenesis, it may also inhibit the bone resorption intensified in these patients, and thus slow down the disease process [44].

Although the assessment of IL-18 in gingival tissue in our study is limited by the number of cases examined, it appears that the expression of IL-18 in the gingiva of patients with periodontitis may be the result of chronic inflammation and the action of other pro-inflammatory factors. IL-18 is a pleiotropic cytokine that is part of a complex inflammatory cascade present in periodontitis and may exert both pro-inflammatory and protective effects. IL-18 may stimulate the process of osteogenesis and it may also inhibit bone resorption intensified in patients with periodontal disease, and thus slow down the disease process. It seems that it depends on the target tissue, the intensity of the inflammation, and the presence of other proinflammatory mediators. Determining the exact role of IL-18 in the pathogenesis of periodontal diseases requires further research.

## 5. Conclusions

The results from our study population suggest that the IL-18 rs187238 and rs1946518 polymorphisms are not significant factors influencing the risk of periodontal disease. However, in patients with the *IL18* rs1946518 GG genotype, we observed increased values of BoP and PPD, compared to subjects with the TT genotype. Additionally, in gingival tissue of patients with periodontitis, decreased expression of IL-18 was observed. The gingival expression of IL-18 in patients with periodontitis correlated positively with number of remaining teeth. The above results suggest that IL-18, in addition to its pro-inflammatory effects in periodontal disease, may also exhibit protective properties.

## Figures and Tables

**Table 1:** The clinical periodontal parameters of the studied subjects.

Parameter	Controls (n = 156)	Periodontitis Patients (n = 200)
SEX (M/F)	54/102	84/116
AGE (years, mean ± SD)	45.40 ± 10.18	49.85 ± 8.71
API (%, mean ± SD)	35.74 ± 20.10	72.98 ± 21.03
BoP (%, mean ± SD)	6.64 ± 11.41	57.66 ± 25.45
PPD (mm, mean ± SD)	1.63 ± 0.54	4.36 ± 2.32
CAL (mm mean ± SD)	0.41 ± 1.18	5.04 ± 2.41

API—approximal plaque index; BoP—bleeding on probing; PPD—periodontal probing depth; CAL—clinical attachment level.

**Table 2:** The distribution of the *IL-18* rs187238 and rs1946518 genotypes in periodontitis patients (PD patients) and the control group.

	PD Patients		Control Group		*p* ^a^		*p* ^b^	OR (95% CI)
	n	%	N	%				
***IL18*** **rs187238**								
**genotype**								
CC	101	50.50	80	51.28	0.79	GG + CG vs. CC	0.92	1.03 (0.68–1.57)
CG	78	39.00	63	40.39		GG vs. CG + CC	0.59	1.29 (0.63–2.67)
GG	21	10.50	13	8.33		GG vs. CC	0.58	1.28 (0.60–2.71)
						CG vs. CC	1.00	0.98 (0.63–1.53)
						GG vs. CG	0.57	1.31 (0.61–2.81)
***IL18*** **rs187238**								
**allele**								
C	280	70.00	223	71.47				
G	120	30.00	89	28.53		G vs. C	0.68	1.07 (0.78–1.49)
***IL18*** **rs1946518**								
**genotype**								
GG	70	35.00	52	33.33		TT + TG vs. GG	0.82	0.93 (0.60–1.44)
TG	94	47.00	83	53.21	0.39	TT vs. TG + GG	0.31	1.41 (0.79–2.53)
TT	36	18.00	21	13.46		TT vs. GG	0.52	1.27 (0.67–2.43)
						TG vs. GG	0.48	0.84 (0.53–1.34)
						TT vs. TG	0.22	1.51 (0.82–2.80)
***IL18*** **rs1946518**								
**allele**								
G	234	58.50	187	59.94				
T	166	41.50	125	40.06		T vs. G	0.70	1.06 (0.79–1.44)

^a^ χ^2^ test; ^b^ Fisher exact test; *IL18* rs187238, HWE: examined group *p* = 0.31, control group *p* = 1.00; *IL18* rs1946518, HWE: examined group *p* = 0.66, control group *p* = 0.24.

**Table 3:** The distribution of the *IL-18* rs187238 and rs1946518 genotypes in periodontitis patients (PD patients) and the controls in the non-smoker group.

	PD Patients		Control Group		*p* ^a^		*p* ^b^	OR (95% CI)
	(Non-Smokers)		(Non-Smokers)					
	n	%	n	%				
***IL18*** **rs187238**								
**genotype**								
CC	62	47.69	63	50.81	0.47	GG + CG vs. CC	0.71	1.13 (0.69–1.85)
CG	54	41.54	53	42.74		GG vs. CG + CC	0.27	1.75 (0.71–4.33)
GG	14	10.77	8	6.45		GG vs. CC	0.25	1.78 (0.70–4.54)
						CG vs. CC	1.00	1.04 (0.62–1.74)
						GG vs. CG	0.35	1.72 (0.67–4.43)
***IL18*** **rs187238**								
**allele**								
C	178	68.46	179	72.18				
G	82	31.54	69	27.82		G vs. C	0.38	1.20 (0.82–1.75)
***IL18*** **rs1946518**								
**genotype**								
GG	43	33.08	41	33.06		TT + TG vs. GG	1.00	1.00 (0.59–1.69)
TG	64	49.23	69	55.65	0.32	TT vs. TG + GG	0.16	1.69 (0.83–3.46)
TT	23	17.69	14	11.29		TT vs. GG	0.32	1.57 (0.71–3.45)
						TG vs. GG	0.68	0.88 (0.51–1.53)
						TT vs. TG	0.14	1.77 (0.84–3.74)
***IL18*** **rs1946518**								
**allele**								
G	150	57.69	151	60.89				
T	110	42.31	97	39.11		T vs. G	0.47	1.14 (0.80–1.63)

^a^ χ^2^ test; ^b^ Fisher exact test.

**Table 4:** The distribution of the *IL-18* rs187238 and rs1946518 genotypes in periodontitis patients (PD patients) and the controls in the smoker group.

	PD Patients		Control Group		*p* ^a^		*p* ^b^	OR (95% CI)
	(Smokers)		(Smokers)					
	n	%	n	%				
***IL18*** **rs187238**								
**genotype**								
CC	39	55.71	17	53.13	0.71	GG + CG vs. CC	0.83	0.90 (0.39–2.09)
CG	24	34.29	10	31.25		GG vs. CG + CC	0.51	0.60 (0.18–2.06)
GG	7	10.00	5	15.62		GG vs. CC	0.51	0.61 (0.17–2.20)
						CG vs. CC	1.00	1.05 (0.41–2.66)
						GG vs. CG	0.49	0.58 (0.15–2.28)
***IL18*** **rs187238**								
**allele**								
C	102	72.86	44	68.75				
G	38	27.14	20	31.25		G vs. C	0.62	0.82 (0.43–1.57)
***IL18*** **rs1946518**								
**genotype**								
GG	27	38.57	11	34.38		TT + TG vs. GG	0.83	0.83 (0.35–2.00)
TG	30	42.86	14	43.75	0.89	TT vs. TG + GG	0.79	0.82 (0.29–2.29)
TT	13	18.57	7	21.87		TT vs. GG	0.77	0.76 (0.24–2.40)
						TG vs. GG	0.81	0.87 (0.34–2.25)
						TT vs. TG	1.00	0.87 (0.28–2.65)
***IL18*** **rs1946518**								
**allele**								
G	84	60.00	36	56.25				
T	56	40.00	28	43.75		T vs. G	0.65	0.86 (0.47–1.56)

^a^ χ^2^ test; ^b^ Fisher exact test.

**Table 5:** The associations between the selected baseline clinical parameters (mean values ± SD) and *IL-18* rs187238 genotypes.

	CC (n = 101)	CG (n = 78)	GG (n = 21)	CC vs. CG *	CC vs. GG *	CG vs. GG *	CC vs. CG + GG *	GG vs. CG + CC *
Age (years)	50.76 ±8.58	49.08 ±8.32	48.29 ±10.57	0.16	0.16	0.57	0.09	0.28
BMI	26.16 ±2.93	26.14 ±3.05	26.30 ±3.22	0.87	0.99	0.92	0.89	0.96
NRT	25.00 ±4.66	24.28 ±5.25	23.29 ±5.41	0.31	0.20	0.42	0.19	0.26
API (%)	75.27 ±19.20	72.05 ±21.04	70.71 ±20.22	0.30	0.34	0.75	0.23	0.48
BoP (%)	60.17 ±25.21	56.87 ±26.00	51.43 ±20.18	0.39	0.11	0.30	0.19	0.15
PPD	4.67 ±1.23	4.47 ±1.17	4.48 ±1.10	0.22	0.43	0.90	0.19	0.69
CAL	5.11 ± 1.54	4.96 ± 1.63	5.19 ± 1.32	0.43	0.73	0.40	0.59	0.55

NRT—number of remaining teeth; API—approximal plaque index; BoP—bleeding on probing; PPD—periodontal probing depth; CAL—clinical attachment level; * Mann–Whitney U test.

**Table 6:** The associations between the selected baseline clinical parameters (mean values ±SD) and *IL-18* rs1946518 genotypes.

	GG (n = 70)	TG (n = 94)	TT (n = 36)	GG vs. TG *	GG vs. TT *	TG vs. TT *	GG vs. TG + TT *	TT vs. TG + GG *
Age (years)	49.66 ±9.14	50.62 ±7.61	48.19 ±10.41	0.54	0.35	0.11	0.90	0.16
BMI	26.13 ±3.04	26.33 ±3.02	25.82 ±2.86	0.86	0.53	0.43	0.92	0.44
NRT	24.93 ±4.67	24.57 ±5.04	23.69 ±5.47	0.63	0.29	0.39	0.44	0.30
API (%)	75.54 ±18.08	72.72 ±20.34	71.75 ±22.87	0.40	0.49	0.98	0.36	0.75
BoP (%)	62.63 ±25.19	56.54 ±26.13	52.61 ±20.87	0.53	0.03	0.34	0.05	0.11
PPD	4.68 ±1.12	4.62 ±1.30	4.22 ±0.98	0.40	0.03	0.12	0.14	0.04
CAL	5.13 ± 1.52	5.08 ± 1.64	4.87 ± 1.36	0.83	0.53	0.66	0.69	0.57

NRT—number of remaining teeth; API—approximal plaque index; BoP—bleeding on probing; PPD—periodontal probing depth; CAL—clinical attachment level; * Mann–Whitney U test.

**Table 7:** The associations between the results of therapy and *IL-18* rs187238 genotypes.

	CC (n = 101)	CG (n = 78)	GG (n = 21)	CC vs. CG *	CC vs. GG *	CG vs. GG *	CC vs. CG + GG *	GG vs. CG + CC *
Patients with clinical improvement after subgingival instrumentation with antibacterial treatment.	82	64	16	0.96	0.86	0.84	0.98	0.84
Patients treated with open flap debridement	10	7	2	0.85	0.72	0.72	0.85	0.75

* Mann–Whitney U test.

**Table 8:** The associations between the results of therapy and *IL-18* rs1946518 genotypes.

	GG (n = 70)	TG (n = 94)	TT (n = 36)	GG vs. TG *	GG vs. TT *	TG vs. TT *	GG vs. TG + TT *	TT vs. TG + GG *
Patients with clinical improvement after subgingival instrumentation with antibacterial treatment.	59	76	27	0.86	0.71	0.81	0.77	0.72
Patients treated with open flap debridement	7	9	3	0.93	0.80	0.89	0.61	0.94

* Mann–Whitney U test.

**Table 9:** Correlations between IL-18 gingival expression and the selected clinical parameters in patients with periodontitis.

Parameter	r	*p*
Age	−0.037	0.89
NRT	0.549	0.04
API	−0.378	0.18
BoP	−0.158	0.58
PPD	−0.239	0.41
CAL	−0.398	0.15

NRT—number of remaining teeth; API—approximal plaque index; BoP—bleeding on probing; PPD—periodontal probing depth; CAL—clinical attachment level.

**Table 10:** Correlations between IL-18 gingival expression and the selected clinical parameters in the control group.

	r	*p*
Age	−0.131	0.75
NRT	0.077	0.85
API	0.325	0.43
BoP	0.365	0.37
PPD	0.214	0.61
CAL	0.407	0.31

NRT—number of remaining teeth; API—approximal plaque index; BoP—bleeding on probing; PPD—periodontal probing depth; CAL—clinical attachment level.

## Data Availability

Not applicable.
